# The effect of regular physical activity on bone mineral density in post-menopausal women aged 75 and over: a retrospective analysis from the Canadian multicentre osteoporosis study

**DOI:** 10.1186/1471-2474-14-253

**Published:** 2013-08-23

**Authors:** Jeffrey M Muir, Chenglin Ye, Mohit Bhandari, Jonathan D Adachi, Lehana Thabane

**Affiliations:** 1 Department of Clinical Epidemiology and Biostatistics, McMaster University, Hamilton, ON, Canada; 2 PhD Candidate, Clinical Epidemiology and Biostatistics, McMaster University, Hamilton, ON, Canada; 3 Department of Surgery, McMaster University, Hamilton, ON, Canada; 4 Department of Medicine, McMaster University, Hamilton, ON, Canada; 5 c/o 3228 South Service Rd, Ste. 206, Burlington, ON L7N 3H8, Canada

**Keywords:** Osteoporosis, Physical activity, Bone mineral density, Post-menopausal

## Abstract

**Background:**

Physical activity is known to benefit many physiological processes, including bone turnover. There are; however, currently no clinical guidelines regarding the most appropriate type, intensity and duration of activity to prevent bone loss.

**Methods:**

To help address this gap in the literature, we performed a retrospective analysis of data from the Canadian Multicentre Osteoporosis Study (CaMos), a prospective cohort of 9423 adult patients, to determine the relationship between the amount of regular daily physical activity performed and bone mineral density. A total of 1169 female participants aged 75 and over provided information regarding their daily activity levels, including the amount of time spent each week performing physical activity at varying levels of intensity. Multiple and linear regression analyses were used to determine the effect of increasing amounts of this regular physical activity on bone mineral density.

**Results:**

The results indicate that a step increase in the amount of physical activity performed each day resulted in a positive effect on bone mineral density at the hip, Ward’s triangle, trochanter and femoral neck (B = 0.006 to 0.008, p < 0.05). Possible confounding factors such as the use of anti-resorptive therapy, body mass index and age were included in the analysis and suggested that age had a negative effect on bone density while body mass index had a positive effect. Anti-resorptive therapy provided a protective effect against loss of bone density.

**Conclusions:**

The data indicate that a step increase in the amount of daily activity, using simple, daily performed tasks, can help prevent decreases in post-menopausal bone mineral density.

## Background

Osteoporosis is a chronic progressive, multifactorial disease and the most common metabolic bone disease in the United States [1]. Affecting both male and females, it is more common in women, affecting post-menopausal women at a rate of 1 in 4 [2]. It is further estimated that 1 in 3 women and 1 in 5 men with osteoporosis will suffer a consequent fracture in their lifetime [3]. Osteoporosis affects an estimated 10 million patients in the United States [4] and is estimated to be responsible for between 1.5 to 2 million fractures each year [5]. The annual medical costs associated with osteoporosis currently range from USD $14 to 20 billion [4,6-9].

The simplest method of limiting this potentially crushing economic burden is disease prevention. As with many conditions, physical activity is promoted as a potentially beneficial activity. The issue, from a clinical perspective, is that there are no guidelines available regarding the most appropriate type, intensity and duration of physical activity to best provide protection against bone loss and/or fracture. The prevailing opinion regarding exercise and bone mineral density is that unloading of the skeleton induces bone loss [10] while loading promotes increased bone mass [11]. Howe et al. [11] recently completed a large Cochrane review of the effect of physical activity and/or exercise programs on bone density. Their findings indicate that, in general, a small, statistically significant protective effect of exercise on bone density was noted in post-menopausal women as compared with control groups. They investigated many different categories of exercise, including those of differing intensity and focus; however, their findings did not translate into specific recommendations that can be used in clinical practice.

Patient compliance with exercise programs is a notoriously difficult area of clinical practice. Many patients do not follow their clinician’s recommendations and, as a result, risk worsening their condition [12]. One method of combating this is to attempt not to prescribe new, unfamiliar exercises to patients but instead to suggest an increase in their normal activities of daily living.

With these difficulties surrounding the prescription of exercise for at-risk patients in mind, we undertook a study with the objective of determining what relationship, if any, exists between the amount of regular physical activity performed each day and bone mineral density. We sought to answer the research question: In post-menopausal women aged 75 and over, does an increase in the amount of regular physical activity performed, measured in hours per week, have a positive effect on bone density? Because the risk of fracture and the consequences associated with fracture increase with age [13], we limited our study to women aged 75 and over, in an attempt to focus on an at-risk demographic.

## Methods

### Canadian Multicentre Osteoporosis Study (CaMos)

The Canadian Multicentre Osteoporosis Study is a prospective cohort study investigating the incidence and prevalence of osteoporosis in Canada. The study methodology, questionnaire design and validation have been summarized previously [14].

### Study design, population and inclusion/exclusion criteria

**Figure 1:**
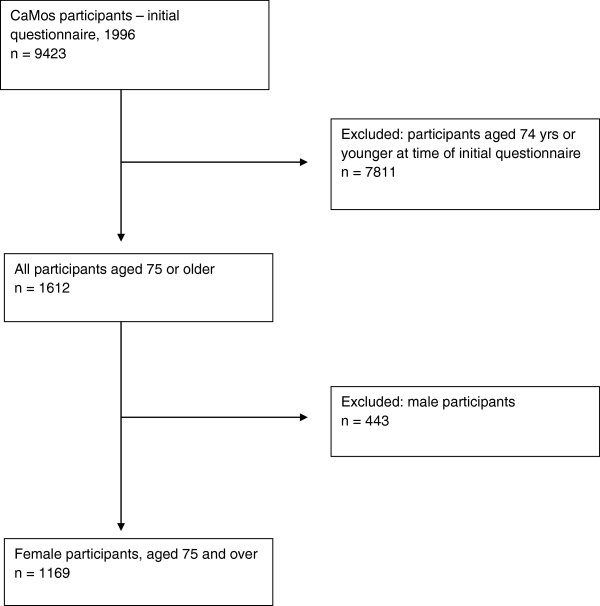
CONSORT flow chart summarizing participant eligibility criteria and resulting number of participants.

The study population for this investigation is a sub-group of the CaMos cohort. Beginning with the initial CaMos cohort of 9423 participants, all participants aged 74 years and younger were deemed ineligible for this study, as were all male participants. As a result, 1169 women aged 75 and over were deemed eligible for this study (see Figure 1).

### Data collection at study entry

Data collected at study entry comprised information gained from baseline questionnaires and from physical examinations. Categories for which information was collected included basic demographic information, physical activity level and participation, bone mineral density, health habits and medications. Physical activity was quantified based on the level of activity and the reported frequency and duration of said activity over the course of the previous 12 months. Anthropometric and demographic details were gathered, including age, sex, study centre, height and weight. Medications identified for analysis included bisphosphonates, hormone replacement therapy and corticosteroids.

### Assessment of physical activity level

Physical activity was assessed through self-reporting on the CaMos questionnaire. The current level of activity for each participant was assessed, i.e. participants were asked to indicate their level of activity, on average, during each week over the previous year. Activities were defined as either “moderate” (housework, brisk walking, golfing, bowling, bicycling on level ground), “strenuous” (e.g. jogging, bicycling up hills, tennis, racquetball, swimming laps) or “vigorous” (e.g. moving heavy furniture, loading or unloading trucks, shoveling, weight lifting). For each level of activity, participants were asked to indicate how many hours per week they spent performing those specific activities. Response options included: never, 0.5-1.0 hours, 2–3 hours, 4–6 hours, 7–10 hours, 11–20 hours, 21–30 hours and 31+ hours. Categories of activity were not mutually exclusive, i.e. a participant could indicate that they took part in moderate, vigorous and strenuous activities over the course of a week if that was their typical pattern of activity.

### Assessment of bone mineral density

Bone mineral density (BMD) was assessed using dual x-ray absorptiometry (DXA) measurements. BMD was measured at five sites: lumbar spine (L1-4), femoral neck, total hip, Ward’s triangle and trochanter. Of the nine CaMos centres, seven used Hologic densitometers (Hologic Inc., Waltham, MA) while the remaining two used lunar densitometers (GE Lunar, Madison, WI). Machines were calibrated daily, as per the manufacturer’s recommendations. Daily monitoring was also used to assess and correct longitudinal drift. Measurements derived from the Lunar instruments were converted to equivalent Hologic values using standard reference formulas [15,16]. Cross-calibration of machines was achieved using an anthropomorphic phantom that was circulated and scanned at each centre.

### Statistical analysis

To determine the relationship between the amount of regular physical activity performed and bone mineral density and fracture rate, two approaches were used.

Initial demographic analysis determined the general features of the cohort, including average age, height, weight, body mass index and similar descriptive statistics. Physical activity levels were analyzed and the cohort examined for frequency patterns.

To determine the effect of physical activity on bone density, regression analysis was used. Linear regression analysis was used to evaluate the effect of varying levels of physical activity at each of the five individual sites. Multiple regression analysis was used to determine the relative effect of increased amounts of regular activity on bone density, taking into account possible confounding factors such as the use of anti-resorptive therapy, body mass index and participant age. All analyses were performed using SPSS 19 (Chicago, IL).

## Results

### Study design, population and inclusion/exclusion criteria

Figure 1 is a CONSORT diagram summarizing the eligibility criteria for the sub-group in question. Following exclusion of ineligible participants, a total of 1169 participants from the initial cohort of 9423 participants were selected for inclusion in the study.

Demographic and anthropometric data for eligible participants was collected. This data is summarized in Table 1. Body mass index (BMI) was calculated as per the World Health Organization. The mean BMI of 26.2 ± 6.5 (median BMI: 26.0) of participants in this study equates to a classification of “overweight”, according to the World Health Organization classification [17].

**Table 1:** Summary of selected demographic and anthropometric characteristics of the study participants

**Characteristic**	**Result (n = 1169)**
Age *(mean ± SD) years*	79.84 ± 4.43
*Age breakdown (number) years*	
75-79	646 (55.3%)
80-84	337 (28.8%)
85-89	143 (12.2%)
90-94	39 (3.3%)
95+	4 (0.3%)
Height in cm *(mean ± SD)*	156.6 ± 6.5
Weight in kg *(mean ± SD)*	64.2 ± 12.1
Body Mass Index (BMI) *(mean ± SD)*	26.2 ± 6.5
Osteoporosis diagnosis? *(number)*	
Yes	506
No	597
Osteoporosis treatment? *(number)*	
Yes	388
No	177
Race	
White	1144^1^
Chinese	14
South Asian *(e.g. East Indian, Pakistani)*	1
Black *(e.g. African, Haitian, Jamaican)*	4
Native/Aboriginal	2^1^
Arab/West Asian	0
Filipino	1
South East Asian *(e.g. Cambodian,*	1
*Indonesian)*	1
Latin American	2
Japanese	0
Korean	0
Medication	
Currently taking antiresorptive medication *(number,%)*	85 (15%)

*SD* standard deviation.

*BMI* body mass index.

^1^ one respondent identified herself as both “white” and “native/aboriginal”.

Participants were asked several questions relating to their level of activity. When asked if they participated in a regular activity program, 47.6% (557/1169) of participants indicated that they did participate in some type of activity program. Participants were asked to indicate how many hours per week they devoted to activities representative of each level of activity – moderate, vigorous or strenuous. Table 2 summarizes the distribution of each level of activity throughout the participants. A vast majority (93.5%) of participants indicated that they took part in regular physical activity at a moderate level over the previous year. Over half (55.3%) of participants indicated that they were active for a minimum of one hour per day, while only 14.5% of participants were active less than an average of 15–20 minutes each day.

**Table 2:** Summary of level of participation in each category of activity for female participants aged 75 years and older over the 12 months prior to study enrollment, in hours/week

	**Level of participation**^**1**^							
	**Never**	**0.5-1 hr**	**2-3 hrs**	**4-6 hrs**	**7-10 hrs**	**11-20 hrs**	**21-30 hrs**	**31+ hrs**
	*Count (%)*	*Count (%)*	*Count (%)*	*Count (%)*	*Count (%)*	*Count (%)*	*Count (%)*	*Count (%)*
Moderate	76 (6.5)	94 (8.0)	163 (13.9)	189 (16.2)	222 (19.0)	219 (18.7)	130 (11.1)	76 (6.5)
Strenuous	1133 (96.9)	15 (1.3)	17 (1.5)	2 (0.2)	1 (0.1)	1 (0.1)	n/r^2^	n/r
Vigorous	1128 (96.4)	31 (2.7)	8 (0.7)	2 (0.2)	n/r	n/r	n/r	n/r

^1^ participants indicated their level of participation for each of moderate, strenuous and vigorous levels.

^2^ no respondents for this category.

Vigorous and strenuous activities were much less represented. The majority of respondents indicated that they had little participation in strenuous or vigorous activity. Indeed, only 36 participants (3.2%) indicated that they took part in any amount of regular strenuous activity. Likewise, only 41 participants (3.6%) responded that they were regularly involved in vigorous activity. The small number of participants that reported taking part in regular activity at a level considered strenuous or vigorous was insufficient to perform statistical analysis. As such, only the effect of varying frequency of regular physical activity at a moderate level (MPA) on bone mineral density was analyzed.

### Possible confounding factors

To address the possible confounding effects of secondary factors known to affect bone density such as age, BMI, race and concurrent medication, data was collected regarding these variables. Data regarding age and BMI is summarized previously. Demographic information regarding race and ethnic background indicate that the vast majority of participants were Caucasian, with fully 97.9% of participants (1144/1169) identifying themselves as “white”. Table 1 provides a summary of the racial and ethnic make-up of the cohort.

Participants were asked to provide information regarding prescribed medications as part of this study. This data included information regarding medications that may or are known to affect bone metabolism, including hormone replacement therapy, corticosteroids and anti-resorptive therapies. For the purposes of this study, the data gathered regarding those participants who were currently taking either anti-resorptive therapy (e.g. bisphosphonates, SERMs) was most important. The results of this are summarized in Table 3. Of the 1169 participants, 150 indicated that they were currently using some type of anti-resorptive medication. One-third (n=50) of that group indicated that they were using bisphosphonates (although the specific bisphosphonate used was not recorded); very few (n=5) indicated that they were currently taking SERMs.

**Table 3:** Distribution of selected pharmacological interventions used by study participants

**Medication**	**Status – currently taking?**			
	**Yes**	**%**	**No**	**%**
Hormone replacement therapy	102	8.7	1067	91.3
Bisphosphonates	50	4.3	1119	95.7
Selective Estrogen Receptor modulators	5	0.4	1164	99.6
Corticosteroids	38	3.3	1131	96.7

### The effect of regular physical activity on bone mineral density

BMD was measured at five locations: the lumbar spine (L1-L4), the femoral neck, trochanter, Ward’s triangle and total hip. To determine the effect of varying amounts of regular physical activity on BMD, linear regression analysis was used. Table 4 summaries the results from this analysis, which revealed positive coefficients for all BMD sites with the exception of the lumbar spine, which was associated with a negative coefficient. All positive coefficients represented statistically significant findings. The results indicate that, for all measured locations, save for the lumbar spine, a step increase in the amount of daily MPA (e.g. increasing activity from 2–3 hours per week to 4–6 hours per week) resulted in a statistically significant increase in BMD. The greatest effect was noted at the total hip, where an increase in bone density of 0.008 g/cm^2^ was noted. The femoral neck and trochanter showed similar improvements (0.006 g/cm^2^ in each locale). At the lumbar spine, a negative effect on bone density was noted, with a decrease of 0.006 g/cm^2^ noted, suggesting that there is a negative relationship between MPA and bone density, although this finding was not statistically significant (B = -0.006 [-0.013, 0.00], p = 0.066).

**Table 4:** Effect of increasing amounts of daily moderate physical activity on bone density at various body sites in study participants

**Site**	**Estimated Coefficient (B)**	**95% Confidence Interval**	**p-value**
Lumbar spine	-0.006	[-0.013, 0.00]	0.066
Femoral neck	0.006	[0.002, 0.010]	0.006*
Total hip	0.008	[0.002, 0.013]	0.004*
Trochanter	0.006	[0.002, 0.011]	0.004*

* statistically significant.

### The effect of possible confounding factors on bone mineral density

To evaluate the effect of possible confounding factors on the relationship between physical activity and bone mineral density, multiple regression analysis was utilized. The results from this analysis are summarized in Table 5. The results of the multiple regression analysis mirror those of the linear regression, where moderate physical activity was associated with a negative coefficient in the lumbar spine. This finding, while not statistically significant, suggests that increasing MPA, when combined with other secondary factors, resulted in a decrease in the likelihood of a protective effect against BMD in the lumbar spine.

**Table 5:** Results from multiple regression analysis of the relative effects of moderate activity and secondary factors on bone mineral density at various body sites

**Variable**	**BMD site**	**Coefficient (B), 95% CI**	**p-value**
Moderate activity	Lumbar spine (L1-4)	-0.006 [-0.013, 0.000]	0.067
	Femoral neck	0.004 [0.000, 0.008]	0.042*
	Total hip	0.006 [0.001, 0.011]	0.019*
	Ward’s triangle	0.004 [-0.001, 0.009]	0.132
	Trochanter	0.005 [0.001, 0.009]	0.018*
Anti-resorptive therapy	Lumbar spine (L1-4)	0.040 [0.006, 0.074]	0.021*
	Femoral neck	0.022 [0.001, 0.043]	0.038*
	Total hip	0.017 [-0.008, 0.042]	0.175
	Ward’s triangle	0.016 [-0.009, 0.041]	0.213
	Trochanter	0.004 [-0.017, 0.025]	0.731
Body mass index	Lumbar spine (L1-4)	0.011 [0.008, 0.014]	0.001*
	Femoral neck	0.008 [0.006, 0.010]	0.001*
	Total hip	0.011 [0.009, 0.013]	0.001*
	Ward’s triangle	0.007 [0.006, 0.009]	0.001*
	Trochanter	0.008 [0.007, 0.010]	0.001*
Age (years)	Lumbar spine (L1-4)	-0.002 [-0.005, 0.002]	0.293
	Femoral neck	-0.005 [-0.007, -0.003]	0.001*
	Total hip	-0.006 [-0.009, -0.004]	0.001*
	Ward’s triangle	-0.005 [-0.008, -0.003]	0.001*
	Trochanter	-0.004 [-0.006, -0.002]	0.001*

* statistically significant.

Information was gathered regarding current medications being used by participants. Of interest to this study was the use of anti-resorptive medications such as hormone replacement therapy (HRT), bisphosphonates and selective estrogen receptor modulators. The data regarding use of specific medications indicated that there was insufficient use of the various anti-resorptive medications to allow individual analysis (see Table 3). As such, all anti-resorptive medications were pooled and those data were utilized in the analysis. Multiple regression analysis indicated that, for all five BMD sites, the use of anti-resorptive therapy produced a positive regression coefficient, although only in the lumbar spine and femoral neck were these findings statistically significant. In the lumbar spine (B = 0.040 [0.006, 0.074], p = 0.021) and femoral neck (0.022 [0.001, 0.043], p = 0.038), increases in moderate physical activity were associated with a protective effect on BMD. In the remainder of BMD sites, regression analysis indicated that there were positive effects on BMD, although not statistically significant (total hip: B = 0.017 [-0.008, 0.042], p = 0.175; Ward’s triangle: 0.016 [-0.009, 0.041], p = 0.213; trochanter: 0.004 [-0.017, 0.025], p = 0.731).

The average (± SD) age for participants was 79.8 (± 4.4) years, with the majority of participants (84.1%) falling within the decade from 75 to 84 years of age. Multiple regression analysis including the participants’ age as a variable produced uniformly negative coefficients, indicating a negative relationship between increasing age and BMD (see Table 5). In all hip-related BMD sites, increasing age was associated with statistically significant negative regression coefficients (femoral neck: B = -0.005 [-0.007, -0.003], p = 0.001; total hip: B = -0.006 [-0.009, -0.004], p = 0.001; Ward’s triangle: B = -0.005 [-0.008, -0.003], p = 0.001; trochanter: B = -0.004 [-0.006, -0.002], p = 0.001). In the lumbar spine, increasing age was also associated with a negative coefficient, although this finding was not statistically significant (B = -0.002 [-0.005, 0.002], p = 0.293).

Body mass index is a widely accepted method of evaluating body fat and body composition. Height, weight and BMI data were collected for each participant in this study. The effect of BMI on BMD was included as part of the multiple regression analysis for this study. The results demonstrate that, for all BMD sites, BMI was associated with a positive and statistically significant coefficient (see Table 5).

## Discussion

Regular physical activity is routinely recommended by clinicians for patients at risk for osteoporosis. There are; however, no specific guidelines for clinicians regarding the type, duration or intensity of physical activity that is most appropriate for these patients. Furthermore, patient compliance regarding exercise programs is generally low in the majority of clinical settings. As such, physical activity in any form becomes important in the prevention of bone loss. The current study sought to determine whether a relationship exists between the amount of regular physical activity performed on a weekly basis and bone mineral density in Canadian women aged 75 and over.

### Physical activity in women aged 75 and over

The vast majority of participants reported some level of involvement in moderate physical activity, i.e. that which could be considered activity over and above the general activity of day-to-day life, such as brisk walking, golfing, housecleaning, etc. Close to three-quarters of participants (71.7%) reported that they are moderately active for at least 4 hours per week. This is an encouraging finding, in light of the fact that, in Canada, up to 64% of female seniors are considered inactive [18] while in the United States, over 60% of senior women were reportedly not meeting the minimum recommendations for regular physical activity (approximately 15–20 minutes daily) [19].

### Effects on bone mineral density

The findings of this study indicate that regular physical activity at a moderate level can help to improve bone density in post-menopausal women, although these improvements were limited largely to the hip region. These findings echo those of similar studies that have shown that the benefits from exercise or physical activity are generally noted in the hip but not in the lumbar spine. Bolton et al. [20] demonstrated in a recent randomized, controlled trial of post-menopausal women that an increase in regular physical activity can have a positive impact on bone mineral density. In their study, over the course of one year, participants took part in a general exercise program that included 60-minute exercise training three times each week, where control participants continued in their normal daily routine. The exercise training group performed tasks including resistance training, moderately intense exercise and training. The authors found that there was a positive (although not statistically significant) effect on bone density in the hip region but a negative (although also not statistically significant) decrease in bone density in the lumbar spine. The measured difference in BMD in the current study closely approximates that of the Bolton study, especially regarding the location of improvements. These findings are likely not unexpected, as the benefit gained from resistance or impact exercise relates largely to the effect of loading on the skeleton [21-23]. The hip joint will absorb the majority of the forces applied during land-based exercise, while the lumbar spine will absorb very little physical force. As such, the majority of exercises are designed to address the hip, an important fact due to the simple fact that the hip, being the structure that absorbs more force during these type of tasks, is also the structure more likely to be damaged (i.e. to suffer a fracture).

The results from the current study contradict the findings of Gerdhem et al. [24], who noted no correlation between previous and current physical activity level and bone mineral density in women up to age 75. The questionnaire used in their study contained 10 questions relating to past and current physical activity level, half of which related to physical activity associated with employment. Only one question related to the current level of physical activity. The more detailed information gained from the CaMos questionnaire regarding current activity level may help explain the contradictory findings. The lack of consistency between physical activity/training programs and duration are discussed by Gerdhem as possible explanations for the contradictory findings in previous studies. Such variations could explain the inconsistencies between the results of these two studies.

The results from this study indicate that there was a statistically significant improvement in bone density associated with a step increase in the amount of moderate physical activity performed on a regular basis. The essential question, then, is: is this improvement clinically important? The most common treatment for osteoporosis are the bisphosphonates. These medications have been shown to induce an average increase of approximately 0.019 g/cm^2^ following a one-year course of treatment [25]. The findings from the current study indicate that the improvements in bone density range from 0.006 g/cm^2^ (for femoral neck, Ward’s triangle and the trochanter) to 0.008 g/cm^2^ (for the total hip). These improvements represent between 30-50% of the improvement expected from bisphosphonate treatment. Warming et al., [26] performed a prospective study to evaluate the normal changes in BMD in the forearm, hip, spine and total body, in otherwise healthy men and women. They used DXA measurements at 2 year intervals in over 500 participants and found that, in women, the only pre-menopausal bone loss was noted at the hip (<0.003 g/cm^2^/year). In women after menopause, though, bone loss ranging from 0.002 g/cm^2^/year to 0.006 g/cm^2^/year was noted in all sites. The greatest post-menopausal bone loss was found in forearm, where 1.2% (0.006 g/cm^2^/year) was lost following menopause, a change that remained constant throughout life. While the changes noted in this study do not meet the level of bisphosphonate treatment, it appears that an increase in the amount of MPA on a daily basis may be enough to offset the normal bone loss that occurs following menopause. If this is indeed the case, the importance of encouraging elderly patients to remain active on a daily basis is underscored.

Several factors were considered possible confounding factors in this study, based on their ability to affect bone mineral density. The results regarding medication use indicate that, perhaps expectedly, the use of anti-resorptive therapy reversed the negative effect on BMD in the lumbar spine and increased the protective effect in each of the other BMD sites, although only the improvements in the lumbar spine and femoral neck were statistically significant. It is not surprising that anti-resorptive therapy counteracted the observed decrease in BMD noted in the lumbar spine and result instead in a positive regression coefficient and a relative increase in BMD.

Other factors considered in this study included race, body mass index (BMI) and participant age. Because race and/or ethnicity are known to impact on bone loss and the incidence of osteoporosis, race was initially intended to be considered as a secondary factor. Analysis of the database; however, indicated that the large majority of participants (97.9%) identified themselves as “white”, which essentially made an examination of the effect of race on bone loss impossible. The relationship between BMI and BMD indicated that increased BMI resulted in a relative protective effect on bone density. These findings support those of several authors [27-29], who have also observed that increased BMI is associated with a lower risk of osteoporosis.

### Limitations

This study has several limitations which prevent the direct application of its findings to clinical settings.

The homogeneity of the cohort with respect to racial and/or ethnicity make-up makes application of the results difficult. With 97.9% of participants identifying themselves as “white”, the ability to determine racial differences is impossible. The CaMos cohort, while sampling from a large proportion of the Canadian population as a whole, does not fully reflect Canadian society as a whole. Indeed, taking the entire CaMos cohort into account, 94.9% of the 9423 participants identified themselves as white. While this may a valuable factor when considering that Caucasian women are at a higher risk of osteoporosis as compared to other racial groups such as blacks or hispanics, the ability to apply the findings to an increasingly racially diverse Canada is limited by these demographics.

The initial plan for this study was to compare physical activity considered part of normal day-to-day activity with more strenuous activity, to determine the relative effects on bone density and fracture rate. The observation that over 96% of the study cohort took part in no vigorous or strenuous activity whatsoever made that analysis impossible. It is unfortunate that more participants were not active to these greater degrees, as it would have better reflected the potential role of exercise in the protection against fracture. However, this finding is mitigated by the fact that beneficial effects were noted simply by increasing the amount of MPA performed each day, which is likely easier in it implementation than incorporating a vigorous exercise program into the routines of elderly patients.

An important factor in this study was the use of anti-resorptive medication by some participants. These medications certainly have a positive effect on bone density; however, their use, in combination with exercise and activity, is an important clinical consideration, especially when clinicians are faced with the choice of prescribing medication for their patients. Of the 1169 participants in this study, only 150 reported currently using anti-resorptive medication. Of those, only 50 were using bisphosphonates, the most common anti-resorptive medication, and a mere 5 were using SERMs. This represents less than 0.5% of the entire study cohort, an amount insufficient to determine a possible confounding effect of anti-resorptive use.

## Conclusions

The results of this study indicate that the amount of regular physical activity at a moderate level in which patients take part each day can have a significant impact on the maintenance of bone density. Because compliance with exercise regimens in the elderly is potentially problematic; this study indicates that, by increasing the amount of normal activity, participants may be able to improve their bone density without having to begin a specific exercise regimen.

## Competing interests

The authors declare that they have no competing interests.

## Authors’ contributions

JMM, JDA, MB and LT designed the study. JMM performed the data collection; JMM and CY performed the data analysis. JMM wrote the manuscript; all authors read and provided revisions to the manuscript. JDA and LT were involved in the conception and creation of the Canadian Multicentre Osteoporosis Study. All authors read and approved the final manuscript.

## Pre-publication history

The pre-publication history for this paper can be accessed here:

http://www.biomedcentral.com/1471-2474/14/253/prepub
